# How short video addiction affects risk decision-making behavior in college students based on fNIRS technology

**DOI:** 10.3389/fnhum.2025.1542271

**Published:** 2025-04-07

**Authors:** Shu Zhang, Shiyi Li

**Affiliations:** ^1^Qilu Normal University, Jinan, China; ^2^Guizhou Normal University, Guiyang, China

**Keywords:** short video addiction, risk decision-making, behavioral addiction, background cues, loss, fNIRS

## Abstract

**Introduction:**

Short video addiction is an emerging form of Internet behavioral addiction characterized by dependent, inappropriate, or excessive use of short video applications. This phenomenon significantly affects decision-making processes and warrants further investigation. Despite the growing prevalence of short video addiction, research on its impact on risky decision-making abilities remains limited. To address this gap, the present study contributes to this critical issue by incorporating neurophysiological data.

**Methods:**

Using functional Near-Infrared Spectroscopy (fNIRS) brain imaging technology and the Balloon Analogue Risk Task (BART) experimental paradigm, this study explored the decision-making behaviors and brain activity of individuals with short video addiction (Individuals with SVA) under varying short video background cues. Adopting a mixed experimental design, the study examined decision-making ability and brain activation as dependent variables across two groups (addiction and control), two outcomes (gain and loss), and two background cue conditions (with and without cues). A total of 45 participants were included in the study: 23 in the addiction group and 22 in the control group.

**Results:**

Behavioral results revealed that individuals with short video addiction demonstrated higher risk-taking tendencies, shorter reaction times, and higher rates of balloon explosions, particularly when exposed to short video cues. Neural data indicated heightened sensitivity to short video cues among individuals with addiction compared to healthy controls. Specifically, the right orbitofrontal cortex (OFC) and right frontopolar area (FPA) exhibited increased activation during risk decision-making with short video cues in the addiction group, while the control group showed no activation in these regions. Additionally, Individuals with SVA displayed greater sensitivity to loss outcomes, with significant OFC activation observed in response to losses but not gains. In risk decision-making scenarios involving short video cues, notable activation was observed in the FPA and left dorsolateral prefrontal cortex (DLPFC) among Individuals with SVA when encountering reward loss. However, no significant differences were observed between the two groups in risk decision-making under the background without short video cue conditions, regardless of gain or loss outcomes.

**Conclusion:**

These findings suggest that Individuals with SVA are more susceptible to the influence of short video-related cues, more sensitive to income loss, and more likely to pursue higher rewards, resulting in higher-risk decisions. The fNIRS results provide critical insights into encouraging healthy short video consumption, informing psychological clinical therapy, and advancing research on addiction-related brain mechanisms.

## 1 Introduction

The rise of new social media platforms has led to a surge in the popularity of short videos, owing to their convenience and shareability (Elbe et al., 2019). These videos cater to the fast-paced nature of modern lifestyles by delivering captivating content in a condensed format (Wang, 2020). Short videos’ quick, fragmented nature can stimulate the brain’s pleasure center, leading to heightened indulgence and cravings in individuals (Tian et al., 2022; Wise and Robble, 2020). This trend is particularly prevalent among college students who meet their learning, self-expression, and social needs. In particular, TikTok has quickly captured the hearts of all age groups and has become the most popular social media platform among millennials in China (Du et al., 2020). The average daily consumption of short videos exceeds 2.5 h, making it the most dominant application in time allocation. The population of short video users in China has surpassed 1.026 billion, representing 95.2% of all internet users across various age groups (China Internet Network Information Center, 2023). However, this behavior can lead to the issue of “short video addiction,” characterized by excessive and compulsive use with potentially adverse effects on mental health, academic performance (Ye et al., 2023a, 2023b; Ye et al., 2024), achievement motivation (Nong et al., 2023), learning avoidance motivation (Ye et al., 2023a, 2023b), well-being (Ye et al., 2022) and social functioning (Zhang et al., 2024; Zhu et al., 2024).

The addictive nature of short-form videos can be attributed to several factors, including the platform’s algorithm-driven content recommendation systems, the brevity and variety of content, and the instant gratification provided by these videos (Liao, 2024; Zhang et al., 2019). These features can lead to prolonged engagement and difficulty in self-regulation, potentially resulting in addiction-like behaviors. As the prevalence of short video addiction increases, it becomes crucial to understand the underlying mechanisms, including depressive symptoms (Xie and Jia, 2021), attention deficits, and compromised decision-making abilities (Liu et al., 2021), among others. Therefore, identifying the risk factors associated with this phenomenon is important to develop effective prevention and intervention strategies (Yang et al., 2022).

Understanding the impact of short video addiction on decision-making is a key area of interest. Decision-making is a crucial cognitive function that influences behavioral tendencies and psychosocial phenomena. It involves evaluating multiple options and selecting the most advantageous ones by considering expected value and utility (Balleine et al., 2007; Hastie, 2001). Decision-making is an optimization process in which individuals balance gains, losses, probabilities, and expectations (Fischhoff and Broomell, 2020). Addictive behaviors can impair cognitive functions, particularly decision-making and inhibitory control (Balogh et al., 2013). Previous studies have demonstrated that individuals with various forms of addiction, such as substance abuse and behavioral addictions, often exhibit altered decision-making patterns, particularly in situations involving risk and uncertainty (Li et al., 2022). Addictive substances can affect the function of the prefrontal cortex, a key area for risk assessment and decision control (Iyer et al., 2022), leading to a decreased ability to evaluate potential negative consequences and a higher risk preference (Balogh et al., 2013). Research on substance addiction has shown that gaming addiction can increase college students’ verbal aggressiveness through decision drift, affecting risk-taking decisions by enhancing impulsivity and reducing inhibitory control compared with non-addicts (Teng et al., 2024). Empirical studies have supported the impact of smartphone addiction on risk-taking behaviors (Khoury et al., 2019; Yuan et al., 2021; Liu et al., 2023). Using functional near-infrared spectroscopy (fNIRS) technology, smartphone addicts have been found to exhibit weaker decision-making abilities and engage in more risk-taking behaviors at high-risk levels. However, the specific effects of short video addiction on risk decision-making remain largely unexplored. Therefore, the first aim of this study was to understand the relationship between short video addiction and risk decision-making across multiple dimensions.

From a decision-theory perspective, individuals exhibit varying behavioral patterns in response to gains and losses. Research on smoking cessation has shown that people are more likely to display impulsive behavior and reduced self-control when faced with losses than when faced with gains (Baumeister and Vonasch, 2015). This cognitive bias can affect how people with an addiction evaluate the consequences of their actions, potentially increasing the likelihood of substance use (Romanowich and Lamb, 2013). Neurobiologically, experiencing loss can influence the brain’s reward system and decision-making processes, suggesting that addictive behaviors may involve the differential processing of gains and losses. Studies using prefrontal electroencephalography (EEG) have demonstrated that losses and gains affect behavioral predictability differently (Telpaz and Yechiam, 2014). For example, gamblers may increase their bets after a loss to recoup their losses, a behavior known as “loss chasing,” a characteristic of problem gambling (Syvertsen et al., 2023). Similarly, internet addicts may become more dependent on the internet when faced with a loss of resources, such as being forced to reduce their online time (Yu et al., 2023). However, it remains unclear whether gains and losses impact short video addiction in different ways. Therefore, the second aim of this study was to investigate the mechanisms underlying the gain and loss of short video addiction.

Background cues play a critical role in the development and maintenance of addictive behaviors. These cues, such as visual, auditory, and olfactory stimuli related to addictive substances or behaviors, can trigger cravings and influence an individual’s choice (Kunas et al., 2022). Research has shown that addiction-related cues activate the brain’s reward system, leading to strong craving responses. For example, the-related cues increase activation in the prefrontal cortex and intensify the desire to smoke (Wanger et al., 2023). This neural response is linked to elevated levels of stress hormones such as cortisol, indicating the physiological basis of addictive behavior. Similarly, drug-related cues induce changes in specific brain regions in a methamphetamine-dependent manner (Yan et al., 2024). These responses are often automatic and difficult to control consciously, reflecting a lack of control over addictive behavior. Cue-induced cravings impact subjective experiences and alter cognitive functions, such as attentional bias and reduced inhibitory control (Waters et al., 2003). Researchers have recently focused on the neural mechanisms of environmental cues in addictive behaviors. Functional magnetic resonance imaging (fMRI) studies have shown that addiction-related cues activate the anterior cingulate cortex and insula, regions associated with reward anticipation and impulse control (Zhang et al., 2020). Background cues in short video addiction include algorithm recommendation systems, content services, platform controls, and user experiences at various levels (Liao, 2024). Short video platforms offer diverse content formats to meet users’ entertainment, social, and learning needs, increasing engagement (Lu et al., 2022). Exploring the relationship between short video addiction behaviors and background cues is the third research objective of this study, with significant theoretical and practical implications based on the research background provided.

To investigate the behavioral and neural factors involved in risk decision-making among individuals with short video addiction (individuals with SVA) compared to healthy controls, this study utilized a modified version of the Balloon Analogue Risk Task (BART), a widely recognized tool for assessing risk-taking behavior (Cazzell et al., 2012). The goal was to uncover potential differences in risk propensity and decision-making processes between the two groups. The BART is a widely used tool in addiction research and has demonstrated sensitivity to various forms of substance and behavioral addictions (Lorains et al., 2014). By adapting this paradigm to incorporate short video-related and no cues, we investigated how exposure to addiction-relevant stimuli may impact risk decision-making in individuals with problematic short video use. This approach allowed us to explore the potential interplay between addiction-related cues and risk-taking behavior, providing insights into the mechanisms underlying addictive behavior in the context of short video use.

To understand the neural mechanisms underlying risk decision-making in short video addiction, we employ functional near-infrared spectroscopy (fNIRS) to measure brain activity during task performance. fNIRS is a noninvasive neuroimaging technique that offers several advantages for studying decision-making processes, including its ability to capture real-time neural activity in naturalistic settings and its relative tolerance to motion artifacts (Carollo et al., 2022). This technique has been successfully used in previous studies to investigate the neural correlates of risk decision-making in various populations (Li et al., 2019), making it suitable for our investigation of short video addiction.

The present study focused on several key brain regions involved in the decision-making and addiction processes. These include the dorsolateral prefrontal cortex (DLPFC), which plays a crucial role in executive functions and cognitive control (Liu et al., 2024), the orbitofrontal cortex (OFC), which is implicated in reward processing and value-based decision-making (Forlani, 2002), and the frontopolar area (FPA), which is associated with complex cognitive processes and prospective thinking (Zhang J. F. et al., 2021). Studies have indicated that the right prefrontal cortex (PFC) exhibits heightened activation levels when faced with beneficial choices in ambiguous decision scenarios, whereas its response remains consistent for both options in risky contexts (Li et al., 2019). Notably, this underscores the importance of the prefrontal cortex in supporting exploratory decision-making, underscoring its crucial involvement in comprehending the decision-making process and mechanisms associated with psychological disorders. Additionally, gambling addicts demonstrate increased activation in the DLPFC when making high-risk, unfavorable decisions compared with low-risk, favorable decisions (Balconi et al., 2018). This indicates that mental addiction is correlated with impaired decision-making capabilities. Hence, examining the functions of prefrontal regions in decision-making is imperative. However, the current understanding of the mechanisms by which short video addiction and decision-making are related remains unclear, particularly regarding the decision-making behavior of individuals with SVA under varying levels of risk. By examining the activation patterns in these regions during risk decision-making tasks, we aimed to identify potential neural markers of short video addiction and their impact on decision-making processes.

In summary, this study investigated the potential differences in decision-making behaviors and brain neural activity between individuals with short video addiction and healthy controls in a risky decision-making context. We modified the BART paradigm and utilized the fNIRS neuroimaging to elucidate variations in risk-taking propensity, decision-making processes, and underlying neural mechanisms. The findings of this study could enhance our understanding of technology-related addiction and guide the development of tailored interventions for short video addiction.

To investigate how short video addiction affects risky decision-making behavior among college students, our study design incorporated several important factors that may influence risky decision-making in this context. First, we manipulated the presence or absence of short video-related cues to investigate how addiction-relevant stimuli might affect decision-making processes. This approach allowed us to examine the potential interaction between cue reactivity and risk-taking behavior, as observed in other forms of addiction (Delaney et al., 2017). Second, we included both gain and loss conditions to explore how the valence of potential outcomes may differentially affect decision-making in individuals with problematic short video use compared to healthy controls. This aspect of our design is supported by previous research suggesting that individuals with addiction may show altered sensitivity to gains and losses.

## 2 Research hypotheses

As previously mentioned, the study proposes three hypotheses:

*Hypothesis 1*. Differential brain neural activity in individuals with SVA: Using fNIRS neuroimaging, it is hypothesized that the activity of the prefrontal cortex (particularly regions associated with impulse control and reward processing) is significantly higher in individuals with SVA compared to healthy controls when exposed to short video-related cues.

*Hypothesis 2*. Differential influence of gain and loss conditions on decision-making behavior: In the risk decision task, individuals with SVA are expected to exhibit a stronger risk appetite under loss conditions than under gain conditions, whereas the healthy control group is anticipated to show minimal differences between gain and loss conditions.

*Hypothesis 3*. Influence of short video-related cues on decision-making behavior in risk-taking scenarios: In risk-taking scenarios, individuals with short video addiction exposed to short video-related cues are expected to demonstrate higher risk preferences (e.g., taking more risks or exhibiting higher balloon burst rates) compared to healthy controls, who are not significantly influenced by these cues.

## 3 Materials and methods

### 3.1 Participants

The study participant size was determined via prior power analysis (PPA), estimating the necessary sample size to guarantee adequate statistical power before the experiment (Liu et al., 2023). Using G*Power 3.1.9 software to estimate the sample size, the statistical power of 0.95 was calculated for 22 participants, with reference to the available literature (partial *η*^2^ > 0.10; Bach et al., 2008; Frühholz et al., 2012). To satisfy the sample adequacy, we recruited 45 participants.

Based on previous research on short video addiction, this study employed the abbreviated version of the College Students’ Short Video Addiction Scale (Qin et al., 2019) to identify participants in both the short video addiction and control groups. Through random sampling, 240 participants (returned questionnaires) were recruited on campus and then categorized using the questionnaire from a university campus in Shandong. A total of 19 invalid data were deleted (incomplete questionnaires). The practical research participants, therefore, numbered 221, with an effective recovery rate of 92.0%. The study included 95 males (43%) and 126 females (57%). There were 42 (19%) first year, 107 (48.4%) second year, 59 (26.7%) third year, and 13 (5.9%) fourth-year students. Finally, a total of 45 participants were recruited, 23 participants (average age 19.6 ± 1.56 years, 11 males and 12 females) participants were selected from the top 27% of the high-scoring of College Students’ Short Video Addiction Scale group as the short video addiction group, and 22 participants (average age 19.7 ± 0.93 years, 5 males and 17 females) were chosen from the bottom 27% of the low-scoring group as the control group.

All the participants were right-handed and had normal hearing. Exclusion criteria included a history of mental illness, head trauma, epilepsy, severe physical illness, or alcohol or drug dependence. Prior to the experiment, the participants were provided with information about the experimental instruments and procedures, signed a written informed consent form, and received compensation. This study was approved by the university Ethics Committee.

### 3.2 Experimental design

This study employed a combination of behavioral measures and functional near-infrared spectroscopy (fNIRS) to investigate the decision-making performance and brain activation levels of short-video addict under different background cues and outcomes using a Balloon Analogue Risk Task (BART) decision-making task paradigm. A mixed experimental design of 2 (group: addiction, control) × 2 (outcome: gain, loss) × 2 (short video background cue: cue, no cue) was utilized, with group as a between-participants variable and the short video background cue and outcome as within-participants variables.

The dependent variables of the study included the number of balloon explosions (NUM), the reaction time (RT) of every trial during the task, and the participants’ brain activation levels as measured by fNIRS.

### 3.3 Experimental tools

College Students’ Short Video Addiction Scale: It is divided into 4 dimensions: withdrawal, loss of control, avoidance and inefficiency, with a total of 22 questions. If the subject answered 5 of the 7 diagnostic questions on the scale, then the subject could be considered to have symptoms of short video addiction, and a higher total score indicates a higher tendency to short video addiction. The reliability coefficient of the total scale was 0.91, and the reliability coefficient of each dimension was between 0.76–0.89 (Qin et al., 2019).

### 3.4 Procedures

This study was conducted in two stages: the scale administration stage and the experimental stage. At the scale administration stage, every participant was required to complete the College Students’ Short Video Addiction Scale (Qin et al., 2019). The participants were divided into two groups based on their scores: one group showing signs of addiction to short videos, and the other serving as the control group. The addiction group comprised individuals from the top 27% of high scorers, while the control group consisted of participants from the bottom 27% of low scorers. At the experimental stage, the participants were asked to make the risk decision-makings by the modified version of the Balloon Analogue Risk Task (BART). Prior to the experiment, participants were informed that the gains in the experiment would be translated into real experimental rewards. Stimulus display and behavioral data acquisition were conducted using E-Prime 3.0. The stimuli were exhibited on a computer monitor with a resolution of 1920 × 1,080. Throughout the experiment, fNIRS cerebral blood oxygen data were collected from the participants’ prefrontal cortex (PFC).

**Figure 1:**
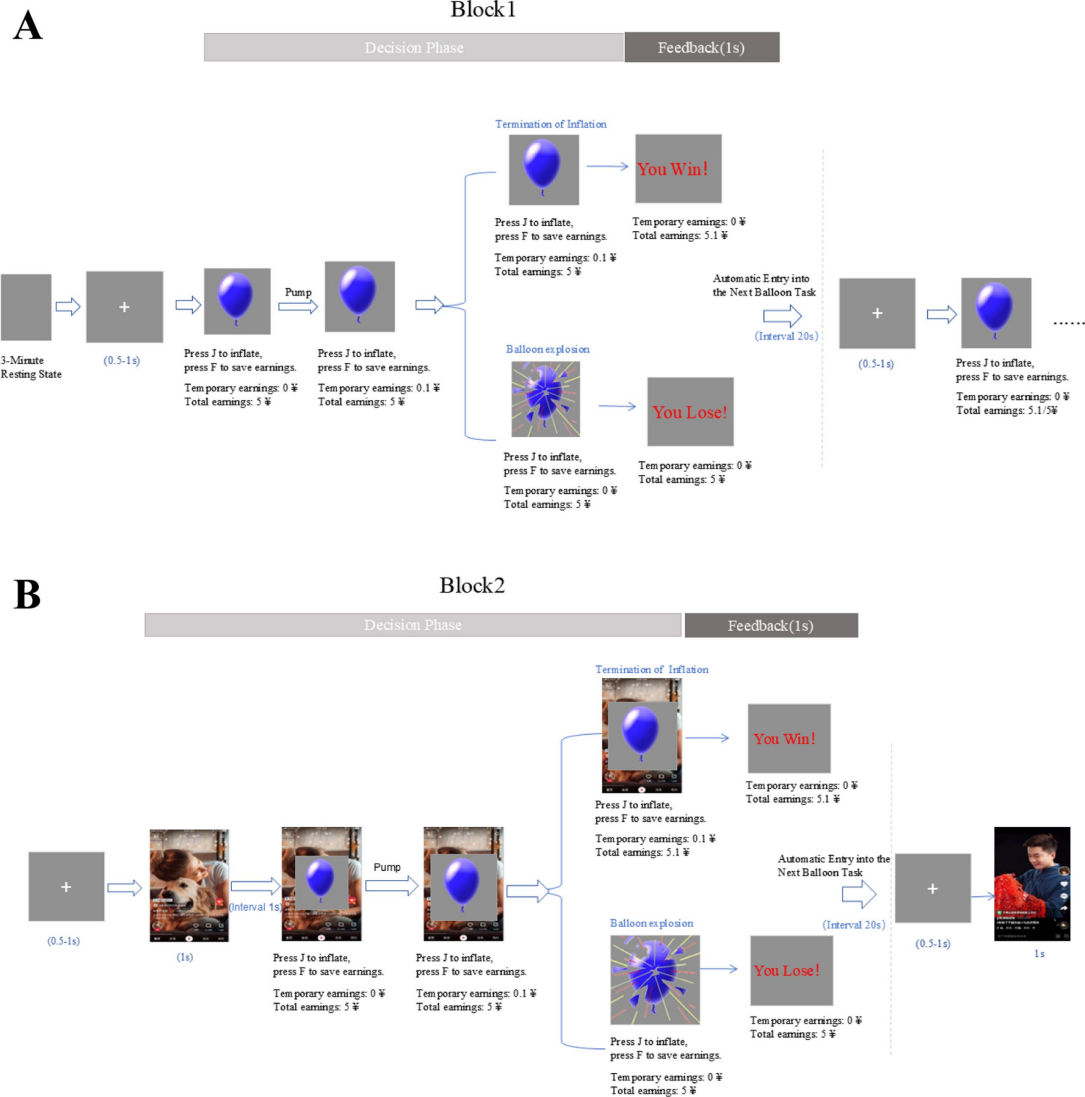
Experimental design procedures. **(A)** The revised BART paradigm with no background images; **(B)** The revised BART paradigm with background images relate to short videos (All the images employed in this study are taken from the Douyin, examples above from users @另一只小叮当 and @剪纸畅杨杨).

The revised BART paradigm, as described by Sui et al. (2024), involved participants being presented with a series of 30 virtual balloons on a computer screen. During each trial, a balloon was displayed, and participants had the option to press J to inflate the balloon or F to cash out. With each pump, both the monetary reward and the probability of explosion increased. Participants received a reward of 10 Chinese cents for each successful pump, but if the balloon exploded, they would lose the accumulated money for that trial. The trial ended when the participant either cashed out or the balloon exploded. The probability of a loss increased with each pump, and the breakpoints occurred randomly without prior information given to the participants (Figure 1A).

The BART procedure in Block 2 closely resembled that of Block 1, but with the short video related pictures shown for 1,000 ms before each balloon and as the background during pumping (Figure 1B). These pictures were randomly selected from a pool of 30 different short video-related pictures. To address the lack of representative stimulus pictures in previous research on short videos, we utilized processed short video pictures as experimental stimuli. Popular short video software and content preferences were identified through questionnaires, relevant content was extracted from short video pictures, and standardized to 227 × 403 pixels. A total of 50 short video cue pictures were gathered, and 80 college students were randomly selected to rate the association between the pictures and short video information on a five-point scale. Thirty short video cue pictures with scores above 3.5 were chosen for the experiment. The experimental design for this study is depicted in Figure 1.

### 3.5 fNIRS data acquisition

**Figure 2:**
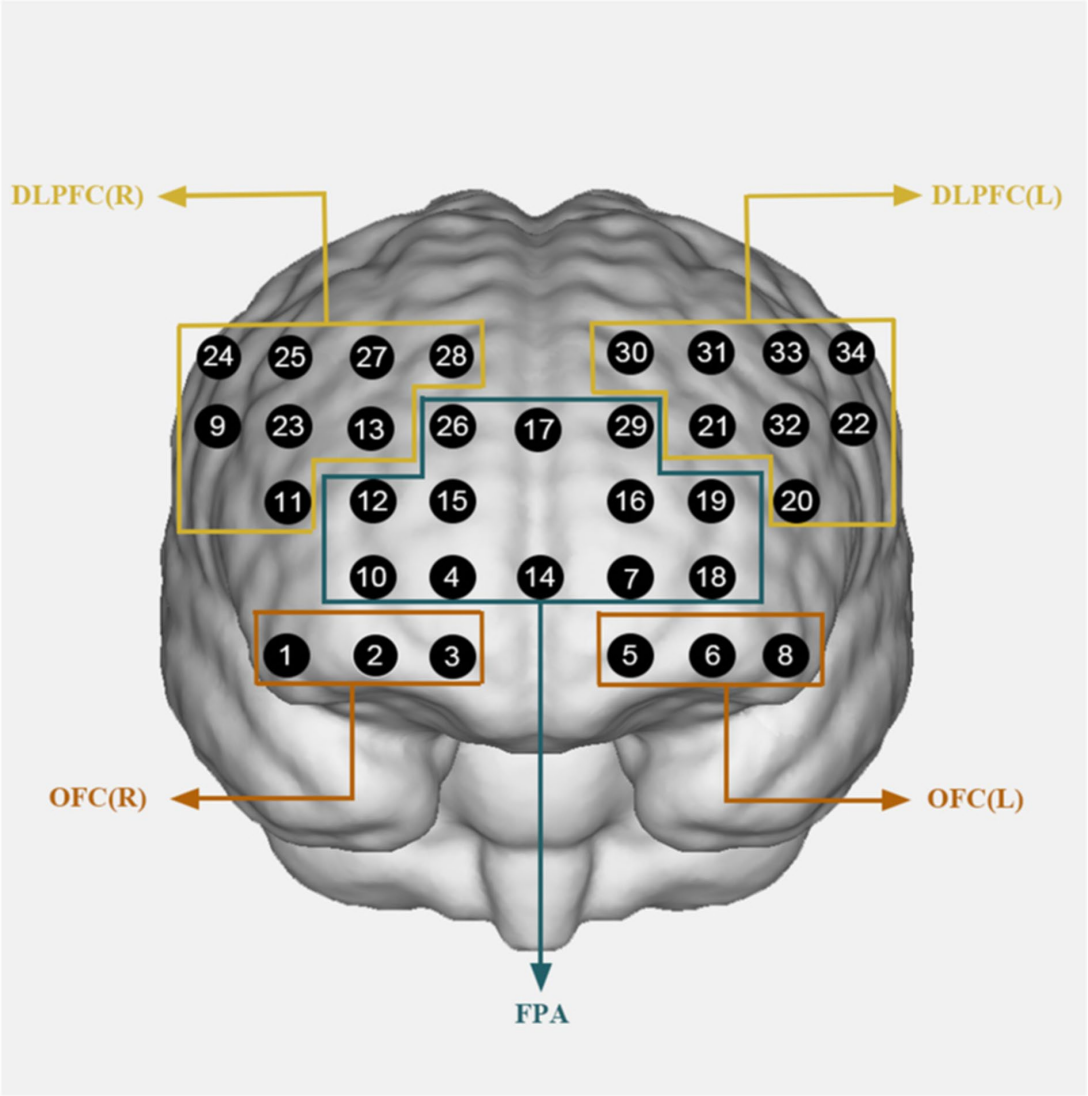
Schematic of arranged fNIRS channels. Dorsolateral prefrontal cortex (DLPFC), frontopolar area (FPA), orbitofrontal area (OFC); R, right; L, left.

In this study, fNIRS data were acquired using a portable near-infrared functional brain imaging device (Huichuang, China). Participants were seated in front of a computer in the laboratory during the data acquisition. The light source detectors were arranged with 12 light sources (Source) and 12 receivers (Detector), forming 34 channels that covered the PFC brain regions explored in this study. The channel positions were determined with reference to the Montreal Neurological Institute (MNI), thus allowing for the selection of specified regions of interest (ROI) in this study. The regions of interest in this study were the right and left dorsolateral prefrontal cortex (DLPFC), frontopolar area (FPA) and orbitofrontal area (OFC). The channels corresponding to each brain region are presented in Figure 2 and Table 1. Spatial alignment information of 34 NIRS channels in the experiment.

**Table 1:** Spatial alignment information of 34 NIRS channels in the experiment.

Location	Channel	SD	Brodmann area	MNI		
				*X*	*Y*	*Z*
DLPFC(R)	9	S7-D12	6-Pre-motor and supplementary motor cortex (0.74)	66	1	27
	11	S8-D7	46-Dorsolateral prefrontal cortex (0.78)	55	37	13
	13	S8-D13	46-Dorsolateral prefrontal cortex (0.77)	48	44	23
	23	S12-D7	45-Pars triangularis Broca’s area (0.37)	58	24	25
			46-Dorsolateral prefrontal cortex (0.36)			
	24	S12-D12	9-Dorsolateral prefrontal cortex (0.51)	57	10	39
	25	S12-D13	9-Dorsolateral prefrontal cortex (0.64)	50	31	37
	27	S13-D13	9-Dorsolateral prefrontal cortex (0.89)	37	46	37
	28	S13-D14	9-Dorsolateral prefrontal cortex (0.84)	16	58	38
DLPFC(L)	20	S10-D10	45-pars triangularis Broca’s area (0.66)	−59	37	13
	21	S10-D15	46-Dorsolateral prefrontal cortex (0.87)	−50	43	23
	22	S11-D16	43-Subcentral area (0.46)	−70	−10	24
	30	S14-D14	9-Dorsolateral prefrontal cortex (0.84)	−12	59	40
	31	S14-D15	9-Dorsolateral prefrontal cortex (0.86)	−35	46	38
	32	S15-D10	45-Pars triangularis Broca’s area (0.41)	−61	17	23
	33	S15-D15	9-Dorsolateral prefrontal cortex (0.61)	−53	28	36
	34	S15-D16	6-Pre-motor and supplementary motor cortex (0.81)	−63	3	36
OFC(R)	1	S2-D2	47-Inferior prefrontal gyrus (1.00)	55	41	−10
	2	S3-D2	10-Frontopolar area (0.60)	42	62	−9
	3	S3-D3	10-Frontopolar area (0.48)	16	72	−9
OFC(L)	5	S4-D3	11-Orbitofrontal area (0.62)	−18	70	−11
	6	S4-D4	11-Orbitofrontal area (0.49)	−45	58	−11
	8	S5-D4	38-Temporopolar area (0.59)	−51	23	−14
FPA	4	S3-D8	10-Frontopolar area (1.00)	32	66	1
	7	S4-D9	10-Frontopolar area (0.98)	−29	68	−1
	10	S8-D2	10-Frontopolar area (0.47)	50	50	2
	12	S8-D8	10-Frontopolar area (0.93)	41	59	13
	14	S9-D3	10-Frontopolar area (0.87)	−1	70	−1
	15	S9-D3	10-Frontopolar area (1.00)	−1	70	−1
	16	S9-D9	10-Frontopolar area (1.00)	−15	72	11
	17	S9-D14	10-Frontopolar area (0.99)	1	64	25
	18	S10-D4	47-Inferior prefrontal gyrus (0.59)	−53	46	−2
	19	S10-D9	10-Frontopolar area (0.92)	−41	59	12
	26	S13-D8	10-Frontopolar area (0.89)	29	60	25
	29	S14-D9	10-Frontopolar area (0.92)	−27	62	26

Thirty-four NIRS channels may cover multiple brain regions. To save space in the paper, this table only lists brain regions with a correlation coefficient greater than 0.4.

### 3.6 Data analysis

As for the behavioral outcomes, a repeated measures ANOVA was performed. The group was the between-groups variable, and the short video background cue and outcome were the within-participants variables. The dependent variables are the number of balloons exploding (NUM) in the simulated balloon explosion mission and the response time (RT).

Regarding the fNIRS results, use the NirSpark software (Huichuang, China) for data pre-processing. (1) Using wavelet-based motion artifact removal (Molavi and Dumont, 2012) to correct the motion artifact of the original optical density data; (2) using 0.2–0.01 Hz filter to filter the data; (3) based on modified Beers–Lambert law, transforming the filtered optical density data into the density change data of HbO and HbR. In this study, Δ[HbO] has a higher SNR than Δ[HbR] and is more sensitive to changes in cerebral blood flow (Tong et al., 2011), so subsequent statistical analysis used Δ[HbO] data.

The general linear model (GLM) was used to calculate the task related *β* value under different conditions, and the *β* value was used as an index to measure the activation of the corresponding brain area. Block averaging was then performed. The statistical analysis was conducted using SPSS 20.0 software (IBM, Somers, United States). The significance level was set at 0.05. Descriptive statistics were reported using mean ± standard deviation. A 2 (group: addiction, control) × 2 (outcome: gain, loss) × 2 (short video background cue: cue, no cue) repeated measures analysis of variance was used to analyze the *β* values of each channel. Greenhouse–Geisser was used to correct the spherical shape, and Bonferroni was used to correct the multiple comparisons. Finally, FDR method was used to correct the multiple comparison of *p* values between channels to further reduce the false positive rate.

## 4 Results

### 4.1 Risky decision-making behavior

**Figure 3:**
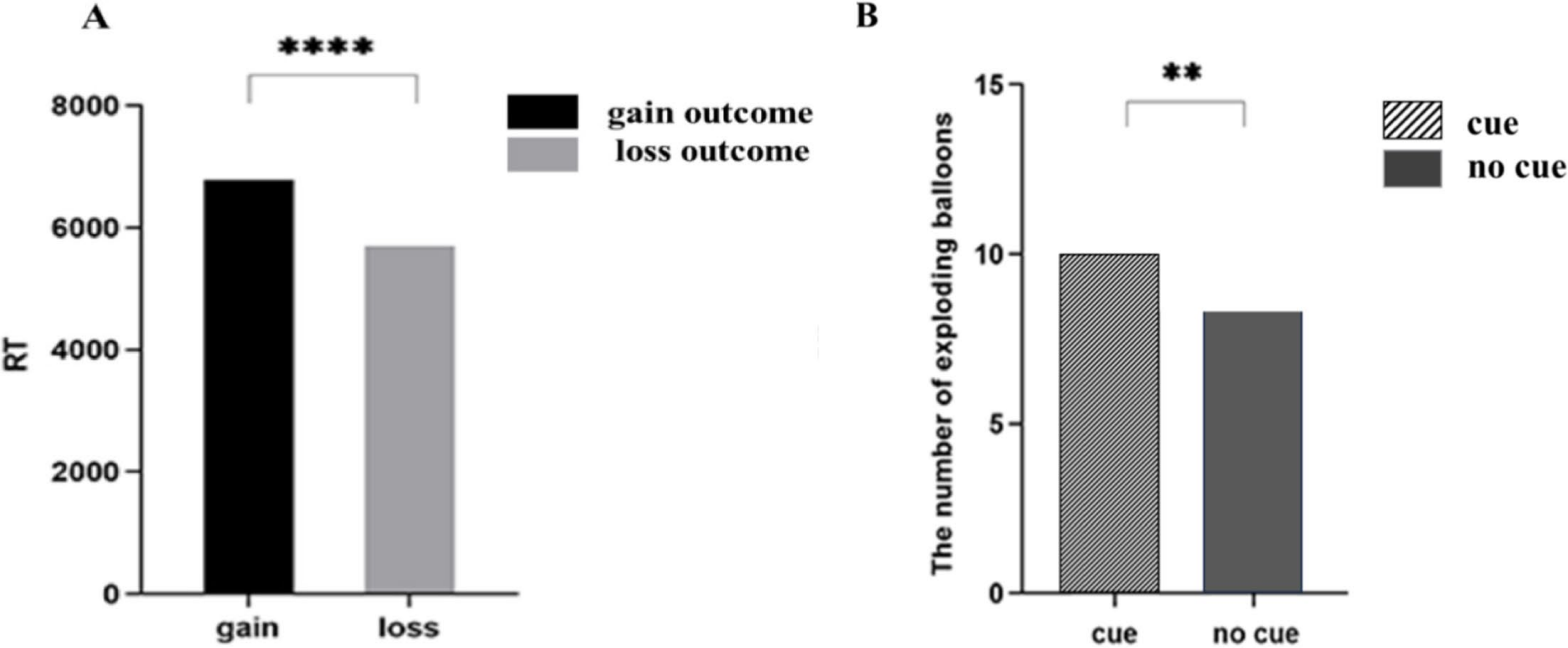
Behavioral results figure. **(A)** The difference in reaction time between gain and loss. **(B)** The difference in the NUM between cue and no cue. ** *p* < 0.01, **** *p* < 0.001.

This experiment aimed to scrutinize the risky decision-making behavior of the participants. The behavioral indicators included the response time (RT) and the number of exploding balloons. A 2 (group: addiction, control) × 2 (outcome: gain, loss) × 2 (short video background cue: cue, no cue) repeated measures ANOVA on RT show that the main effect of the outcome is significant [*F*(1, 42) = 46.866, *p <* 0.01, partial *η*^2^ = 0.533]. Participants exhibited shorter reaction times for the less outcome condition (5827.240 ± 372.842 ms) compared to the gain outcome condition (6905.498 ± 430.416 ms). No significant group differences were found between the short video addiction group and the control group. The results are depicted in Figure 3A.

The number of exploding balloons revealed a significant main effect of the short video background cue was significant [*F*(1, 42) = 8.514, *p <* 0.01, partial *η*^2^ = 0.172], with the findings presented in and Figure 3B. The result showed that the number of exploding balloons with short video picture-related cues (10.136 ± 0.550 units) was more than that without cues (8.398 ± 0.569 units). No significant interactions were observed among the remaining levels. The results are depicted in Figure 3B.

### 4.2 fNIRS results

This study employed a 34-channel, repeated-measures ANOVA of *β* values derived from GLM estimation for different conditions in various groups. The experimental design was a 2 (group: addiction, control) × 2 (outcome: gain, loss) × 2 (short video background cue: cue, no cue) for HbO. The findings are as follows.

#### 4.2.1 The main effect of the outcome

In Channel 2, 4, 6, 7, 10, 20, 21, 22, 29, 30, 31, 32 and 33, the main effect of the outcome was significant. The loss outcome condition elicited more cortical activity than the gain condition. Significant differences in *β* values were observed in the DLPFC, FPA, and OFC regions under the loss condition (refer to Table 2).

**Table 2:** The significance of *β* values in outcome.

Channel	Source-detector	Brodmann area-location	*F*	*p*_FDR_	*η*_p_^2^	df	The *β* of gain (M ± SD)	The *β* of loss (M ± SD)
CH2	S3-D2	10-Frontopolararea	12.884^**^	0.001	0.231	43	−0.035 ± 0.007	0.000 ± 0.010
CH4	S3-D8	10-Frontopolararea	10.414^**^	0.002	0.195	43	−0.027 ± 0.006	−0.005 ± 0.007
CH6	S4-D4	11-Orbitofrontalarea	12.672^**^	0.001	0.228	43	−0.025 ± 0.005	0.003 ± 0.007
CH7	S4-D9	10-Frontopolararea	9.489^**^	0.004	0.181	43	−0.010 ± 0.004	0.008 ± 0.005
CH10	S8-D2	10-Frontopolararea	7.389^**^	0.009	0.147	43	−0.036 ± 0.005	−0.015 ± 0.008
CH20	S10-D10	45-parstriangularisBroca’sarea	8.455^**^	0.006	0.164	43	−0.019 ± 0.005	−0.001 ± 0.007
CH21	S10-D15	46-Dorsolateralprefrontalcortex	8.755^**^	0.005	0.169	43	−0.023 ± 0.004	−0.008 ± 0.005
CH22	S11-D16	43-Subcentralarea	9.831^**^	0.003	0.186	43	−0.016 ± 0.004	0.001 ± 0.006
CH29	S14-D9	10-Frontopolararea	10.279^**^	0.003	0.193	43	−0.013 ± 0.003	0.002 ± 0.004
CH30	S14-D14	9-Dorsolateralprefrontalcortex	9.906^**^	0.003	0.187	43	−0.016 ± 0.003	−0.004 ± 0.004
CH31	S14-D15	9-Dorsolateralprefrontalcortex	9.965^**^	0.003	0.184	43	−0.016 ± 0.003	−0.004 ± 0.004
CH32	S15-D10	45-parstriangularisBroca’sarea	8.582^**^	0.005	0.166	43	−0.016 ± 0.005	−0.004 ± 0.005
CH33	S15-D15	9-Dorsolateralprefrontalcortex	11.251^**^	0.002	0.207	43	−0.022 ± 0.004	−0.007 ± 0.005

Note: ** *p*<0.01.

#### 4.2.2 The interaction between group and short video background cue

**Figure 4:**
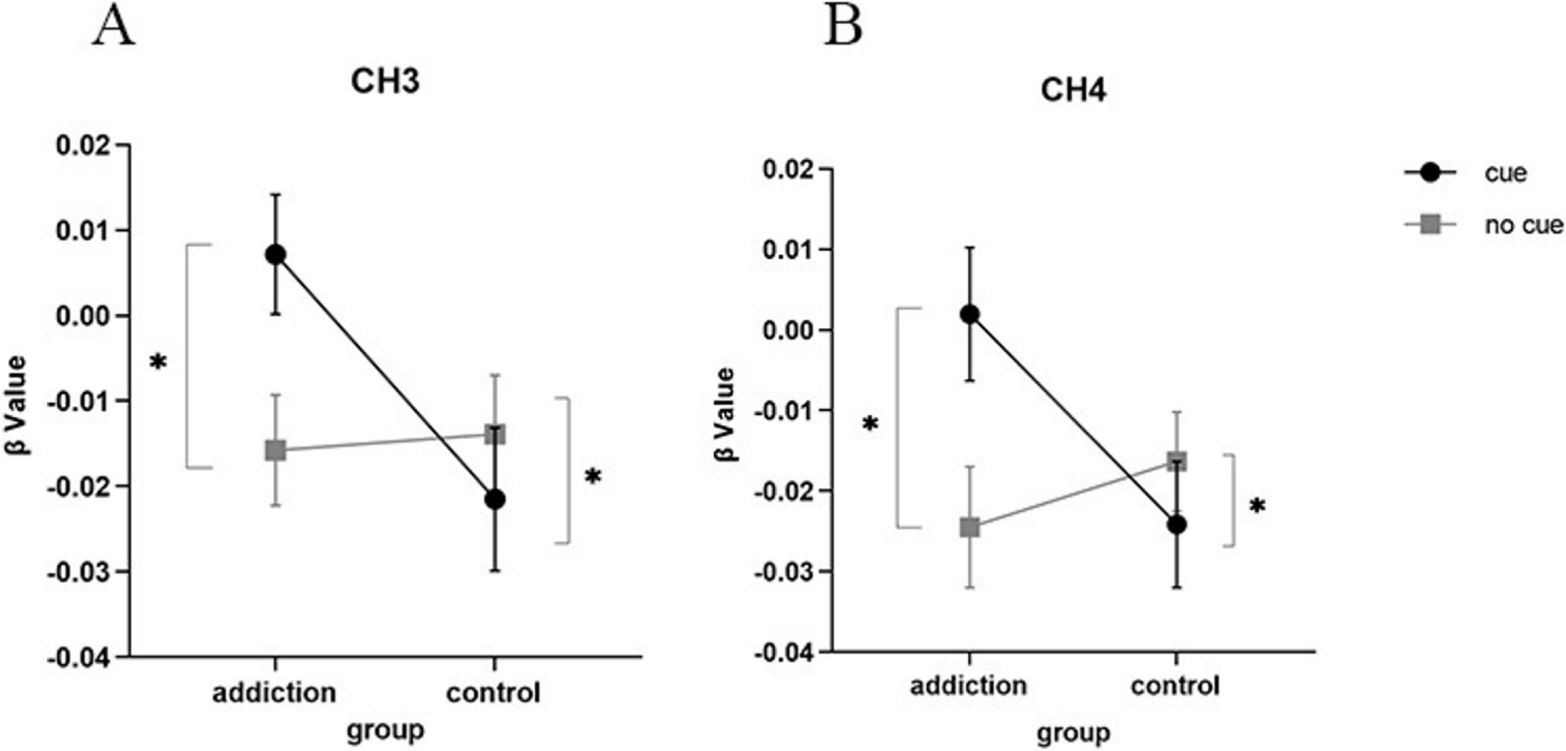
The interaction between group and short video background cue. **(A)** The difference in the Channel 3; **(B)** The difference in the Channel 4. * *p*<0.05.

In Channel 3 and Channel 4, a significant interaction was found between the group and short video background cue [*F*(1, 43) = 4.791, *p* = 0.034, partial *η*^2^ = 0.100 (CH3); *F*(1, 43) = 5.965, *p* = 0.019, partial *η*^2^ = 0.122 (CH4)]. A simple effects analysis showed that the Channel 3 (responding to the frontopolar area), Channel 4 (responding the frontopolar area) of the short-video addict showed a significant increase of HbO concentration than control group, under the short video picture-related cue [*F*(1, 43) = 5.560, *p* = 0.023, partial *η*^2^ = 0.144 (CH3); *F*(1, 43) = 4.601, *p* = 0.038, partial *η*^2^ = 0.097 (CH4)]. While under no cue condition, there was no significant difference observed in Channel 3 and Channel 4 between the two groups (*p* > 0.05). The results are depicted in Figure 4.

#### 4.2.3 The interaction between group and outcome

**Figure 5:**
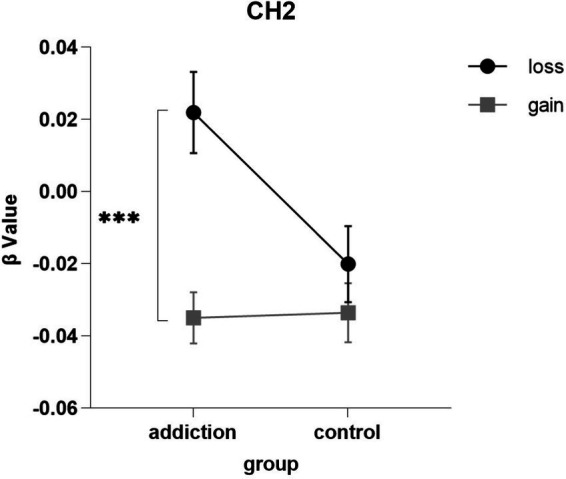
The interaction between group and outcome. *** *p*<0.001.

We found a significant interaction between the group and outcome on Channel 2 [*F*(1, 43) = 4.680, *p* = 0.036, partial *η*^2^ = 0.098], which corresponds to frontopolar area (FPA) on the right. The simple effects analysis revealed that for the short-video addict group, the right FPA was more sensitivity to the loss outcome (0.022 ± 0.014) than the gain condition (−0.035 ± 0.009) in the BART task [*F*(1, 43) = 16.923, *p* < 0.001, partial *η*^2^ = 0.282]. And there was no significant difference between the two levels of outcome in the control group. The results are depicted in Figure 5.

#### 4.2.4 The interaction among group, outcome and short video background cue

**Figure 6:**
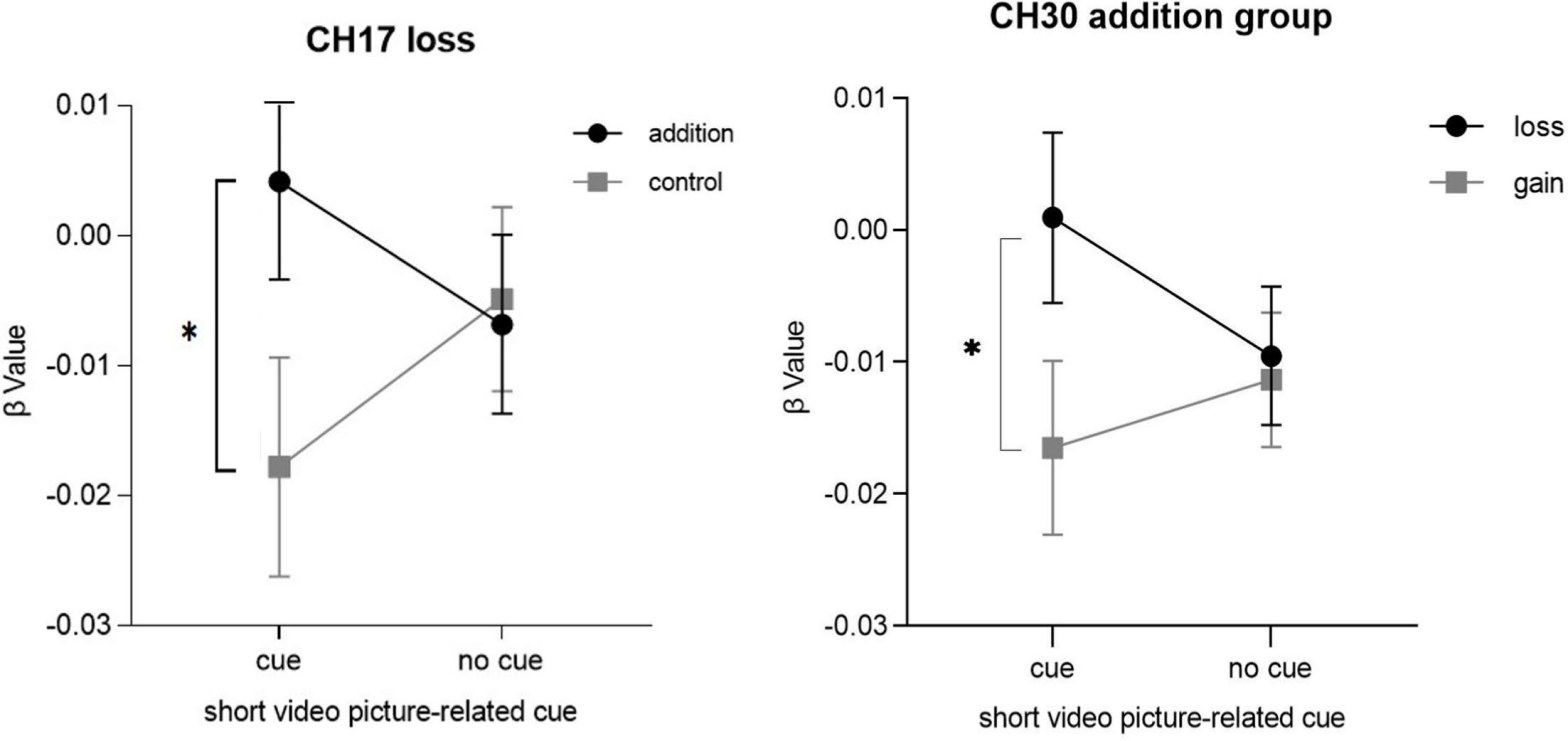
The interaction among group, outcome and short video background cue. * *p*<0.05.

In Channel 17 and Channel 30, a significant interaction was found among group, outcome and short video background cue [*F*(1, 43) = 4.195, *p* = 0.047, partial *η*^2^ = 0.089 (CH17); *F*(1, 43) = 4.134, *p* = 0.048, partial *η*^2^ = 0.088 (CH30)]. In the context of a short video picture-related cue and loss outcome condition, individuals with short video addiction (0.004 ± 0.008) exhibited a significant increase in HbO concentration compared to the control group (−0.018 ± 0.008) when confronted with loss outcomes on Channel17. Channel17 corresponds to the frontopolar area [FPA, *F*(1, 43) = 3.789, *p* = 0.048, partial *η*^2^ = 0.081]. In the short video addiction group and context of a short video picture-related cue condition, individuals with less outcome (0.001 ± 0.006) exhibited a significant increase in HbO concentration compared to the gain outcome condition (−0.017 ± 0.006) on Channel30. Channel30 corresponds to dorsolateral prefrontal cortex [DLPFC, *F*(1, 43) = 7.074, *p* = 0.011, partial *η*^2^ = 0.141]. In the absence of the short video picture-related cues, there was no significant difference between the two participant groups in their responses to loss or gain outcomes. The results are depicted in Figure 6.

**Figure 7:**
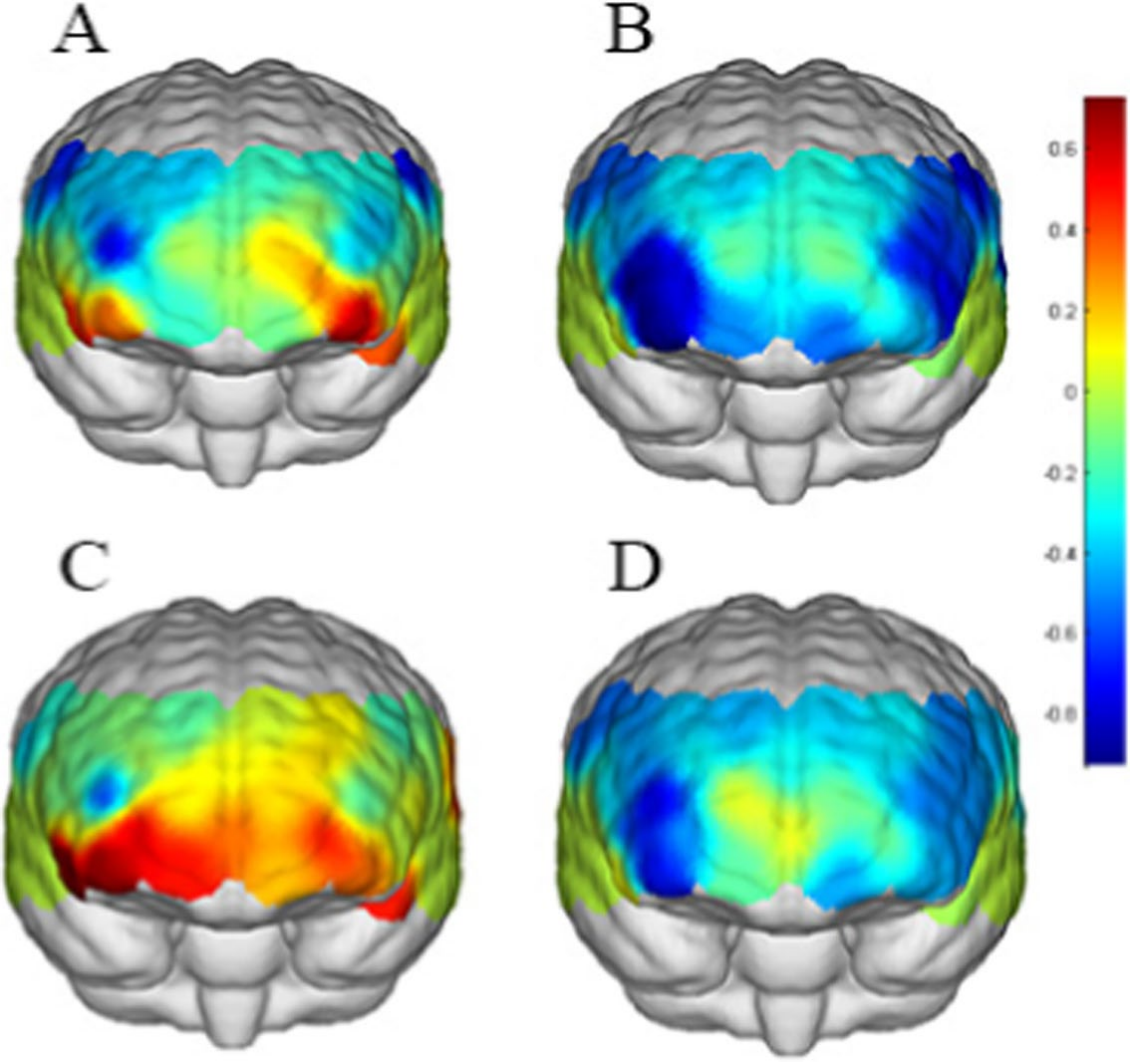
Activation maps of the measured brain regions within the addiction group. **(A)** Represents the addiction group under loss outcome condition with no cue. **(B)** Represents the addiction group under gain outcome condition with no cue. **(C)** Represents the addiction group under loss outcome condition with cue. **(D)** Represents the addiction group under gain outcome condition with cue.

**Figure 8:**
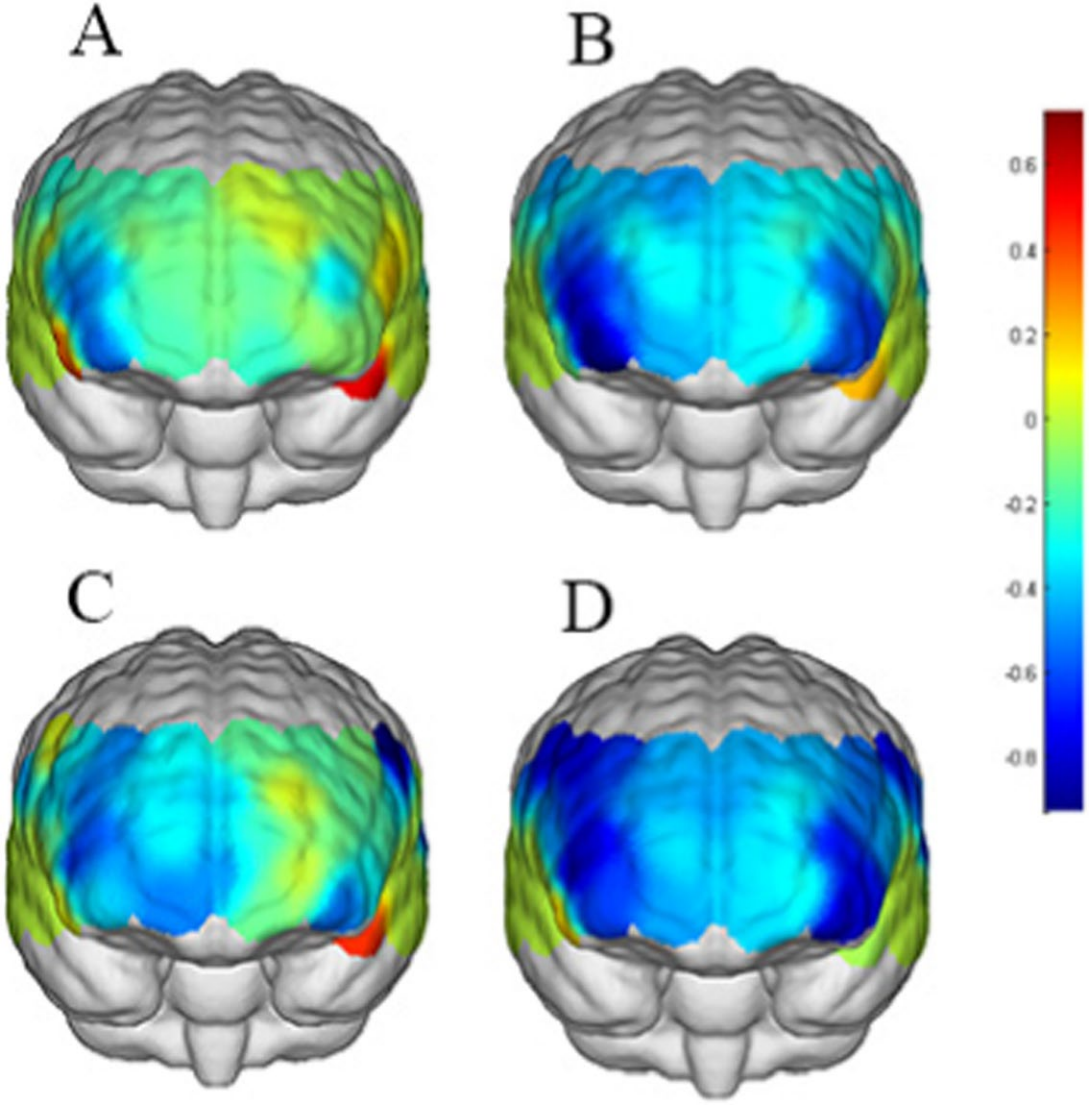
Activation maps of the measured brain regions within the control group. **(A)** Represents the control group under loss outcome condition with no cue. **(B)** Represents the control group under gain outcome condition with no cue. **(C)** Represents the control group under loss outcome condition with cue. **(D)** Represents the control group under gain outcome condition with cue.

The correlated brain activation measurements of the participants are depicted in Figures 7, 8.

#### 4.2.5 Association between fNIRS and behavioral performance

**Figure 9:**
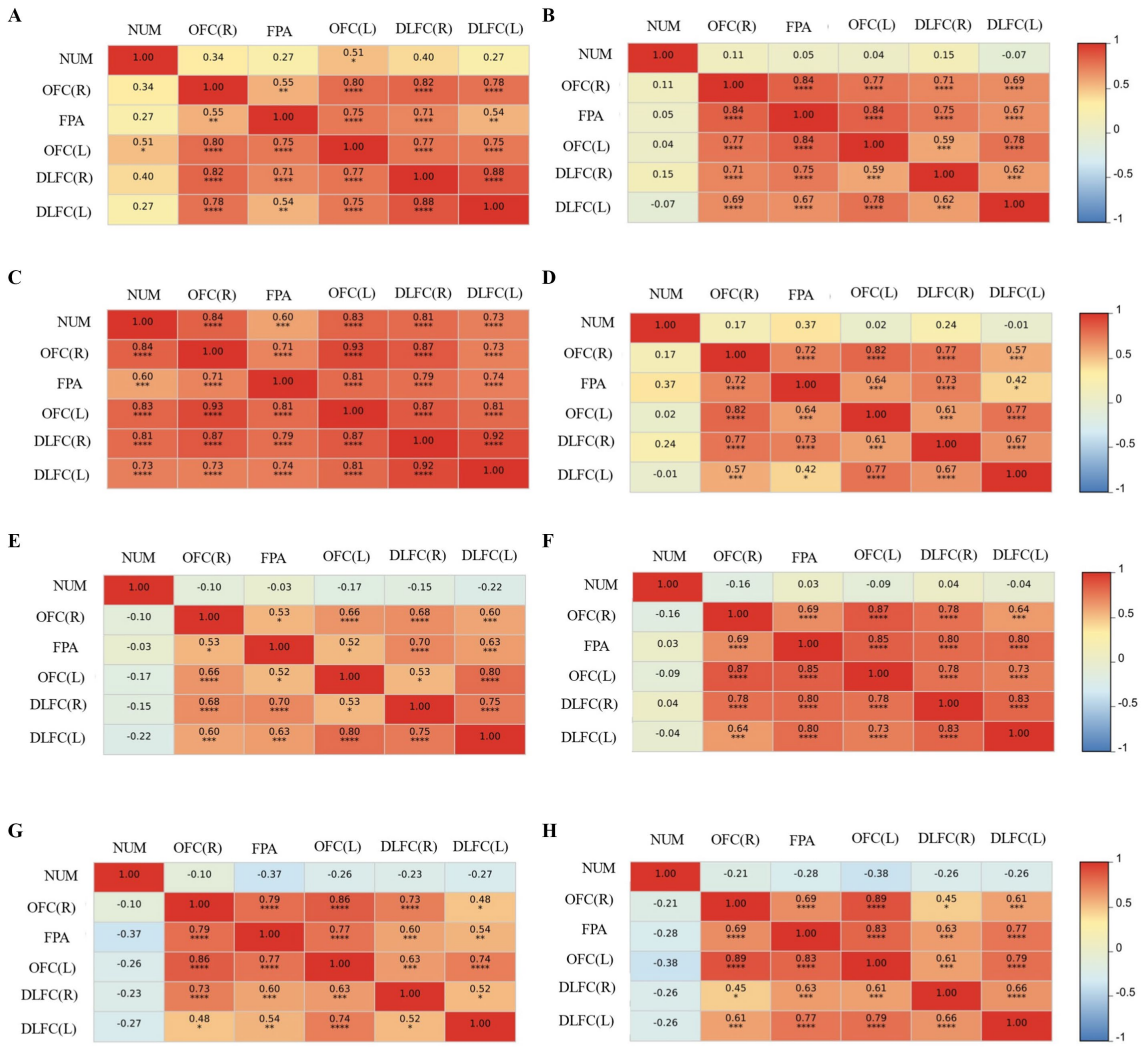
The relationship between each ROI and the number of balloons exploding (NUM). **(A)** SVA under loss outcome without short video picture cue. **(B)** SVA under gain outcome without short video picture cue. **(C)** SVA under loss outcome with short video picture cue. **(D)** SVA under gain outcome with short video picture cue. **(E)** CG under loss outcome without short video picture cue. **(F)** CG under gain outcome without short video picture cue. **(G)** CG under loss outcome with short video picture cue. **(H)** CG under gain outcome with short video picture cue.

Using Pearson’s correlation analysis, we examined the relationship between each ROI and the number of balloons exploding (NUM). The grand-average correlation coefficient matrices between each ROI and the NUM were compared between short video addict and control groups across four conditions (showed in Figure 9). It was found that individuals with SVA had stronger brain activation in all regions when facing losses in a short video background cue condition (*p*-value’s <0.05), while only the activation of the left OFC was positively correlated with the number of exploding balloons in the non-short video background cue condition (*p* < 0.05). In addition, there was no correlation between the number of exploding balloons and other conditions.

## 5 Discussion and conclusion

### 5.1 Discussion

#### 5.1.1 Discussion on behavior mechanisms

The rise of short video platforms has drawn the attention of researchers owing to their impact on user behavior. Short videos as visual cues can influence individuals’ decision-making processes (Smith et al., 2024). Previous studies have indicated that visual cues affect individuals’ risk perceptions and decision-making behaviors (Bougherara et al., 2022). However, there is a lack of discussion on the mechanisms of short video problem use, particularly how short video cues influence the risk decision-making behaviors of different groups. This study aimed to comprehensively analyze the decision-making behaviors and neural activities of individuals with SVA by combining BART with fNIRS technology. This study investigated the impact of short video problem use on college students’ risk decision-making in various scenarios from both behavioral and neural perspectives.

Balloon-based tasks are commonly used to assess individuals’ propensity for risk-taking behaviors (Deng et al., 2023). In BART, reaction time is crucial for evaluating the level of deliberation in decision-making processes (Mahmoodi et al., 2023). Shorter reaction times typically indicate less cognitive processing during decision-making, potentially leading to increased risk-taking behaviors (Toschi et al., 2023). Our study found that participants exposed to short video cues had significantly shorter reaction times than those without cues when faced with gain situations. This suggests that short video cues influenced participants’ decision-making processes. After receiving a reward, participants had to decide whether to continue inflating the balloon for higher rewards or stop to avoid the risk of the balloon bursting, which is a high-risk decision-making scenario. Shorter reaction times with short video cues imply that these cues affect participants’ decisions, prompting them to take more risks in pursuing greater rewards during reward acquisition. Previous research has shown a positive correlation between individuals’ reaction times and tendency to avoid risk in high-risk decision-making contexts (Nishio et al., 2024). However, our study did not find any significant differences between participant groups.

In the BART paradigm, rewards increase as each balloon is inflated, thereby increasing the risk of an explosion. Balloon explosions result in the loss of all rewards, reflecting impulsivity and risk-taking behavior (Doumas et al., 2017). Our study found that participants exposed to short video cues had a higher rate of balloon explosions, indicating a potential influence on risk perception and decision-making. Participants tended to ignore potential dangers and prioritized thrill and enjoyment after viewing the prompts. This shift may be due to prompts that trigger curiosity and desire for adventure. However, there were no significant differences between participant groups, complicating the interpretation of the effects of short video cues. For further analysis, we used functional near-infrared spectroscopy (fNIRS).

#### 5.1.2 Discussion of neural mechanisms

Through near-infrared brain function imaging, it was found that individuals with SVA displayed heightened sensitivity to short video cues compared to healthy controls. Specifically, the right OFC, (Channel 3) and FPA (Channel 4) showed increased activation during risk decision-making with short video cues in individuals with SVA, whereas the control group did not exhibit activation in these brain regions. Furthermore, individuals with SVA also demonstrated greater sensitivity to loss outcomes, with significant activation observed in the OFC (Channel 2) when facing losses but not gains. In risk decision-making scenarios with short video cues, the FPA region (Channel 17) and left DLPFC (Channel 30) were notably activated in individuals with SVA when faced with reward loss. Interestingly, the degree of brain region activation in individuals with SVA in response to loss in the presence of short video cues positively correlated with the number of exploded balloons.

##### 5.1.2.1 Individuals with SVA display specialized neural reactivity to short-video cues in the right OFC and FPA

Individuals with SVA are driven by specific sensitivity to short video cues, as evidenced by significant activation of the right OFC and FPA in the brain. The OFC is crucial for risk/reward decision-making, evaluation of reward values, error processing, and behavioral adjustment (Rolls et al., 2020; Kim et al., 2023). Research suggests that people with addiction often have hyperactive or damaged OFCs, leading to difficulties in evaluating long- and short-term rewards. This altered decision-making may predispose individuals to pursue short-term pleasure despite the long-term consequences (Xu et al., 2024). Additionally, OFC activation is directly related to reward size and pleasure intensity, leading to intense cravings (Gu et al., 2021). Short videos with rich stimuli can trigger pleasure in users, making it difficult for them to disengage from a platform. Repeated exposure to pleasure-inducing cues can lead to hypersensitivity in reward circuits, resulting in intense cravings and potentially driving addictive behaviors. Activation of the OFC may be associated with increased attention to cues in short videos, thus impairing rational decision-making. This heightened response in reward-related neural circuits may impair individuals’ ability to regulate their usage and drive further addictive behavior in short video addiction.

Moreover, the FPA is critical in managing complex behavioral conflicts such as integrating information, planning, future thinking, and social cognition. Activation of the FPA reflects addicts’ efforts to resolve internal conflicts during decision-making, balancing immediate rewards with long-term consequences. Excessive FPA activation may occur during the processing of addiction-related future scenarios and rewards. The overactivation of the FPA in people with addiction is associated with reward expectations and simulations of future scenarios (Falck et al., 2024). Short video platforms utilize personalized recommendations to enhance optimism and exacerbate FPA overactivation, thereby making disengagement challenging. People with addiction tend to overvalue short-term pleasure and underestimate long-term consequences (Estévez et al., 2021). Individuals with SVA exhibit an abnormal attentional bias towards short video cues, prioritizing visual and auditory information related to short videos. Short video platforms use personalized recommendations to fuel optimism and exacerbate FPA overactivation, making it difficult to stop watching. People with addiction overestimate short-term pleasure and underestimate long-term consequences. Although people with addiction may be aware of the negative consequences of long-term addiction to short videos (such as wasted time and decreased attention), the strong impetus for immediate satisfaction often makes it difficult for rational goals to dominate. Therefore, the overactivation of the FPA in individuals with SVA reveals an impairment in their ability to ignore external stimuli that are not conducive to long-term goals. This finding indicates a conflict between immediate gratification and long-term objectives among individuals with SVA.

##### 5.1.2.2 Individuals with SVA exhibit deficiencies in cognitive control with FPA and left DLPFC

Individuals with SVA may have limited cognitive control, with the FPA and left DLPFC playing key roles in decision-making and impulse inhibition. The increased activation of these areas suggests additional cognitive effort in resisting impulses and regulating behavior in individuals with SVA facing potential losses. The FPA in the brain is associated with advanced functions such as cognitive tasks, planning, social cognition, and emotional regulation. In addition, FPA reflects the conflict between short-term gratification and long-term consequences. People with addiction are more sensitive to loss, leading to intense responses to loss situations. FPA activation shows people with addiction struggle between immediate rewards and long-term outcomes as they assess losses and make decisions. Despite facing losses, they may try to predict rewards or adjust their behavior. The DLPFC is crucial for self-control, decision-making, and goal-oriented behavior. An imbalance in DLPFC activity is linked to addiction patterns, including heightened reward sensitivity and impulsivity (Stanković et al., 2023). In this study, individuals with SVA showed left DLPFC activation, indicating their susceptibility to short video content and impaired cognitive control. People with addiction often exhibit heightened sensitivity to immediate rewards in brain regions such as the nucleus accumbens and ventral striatum. In individuals addicted to short videos, this reward system may be altered, leading to increased temptation and emotional responses to such stimuli. When making decisions involving risks and potential losses, the DLPFC plays a crucial role not only in self-control but also in interacting with other regions such as the nucleus accumbens and prefrontal cortex (Chmiel et al., 2023). The development of addiction involves the reward system in the brain, including regions such as the ventral tegmental area, nucleus accumbens, and prefrontal cortex. When individuals experience loss, the DLPFC may be activated to enhance self-control and regulate emotional responses. Research has shown that people with Heroin addiction exhibit heightened cravings when exposed to drug-related cues associated with abnormal DLPFC activation. Similarly, online game addicts show increased attention to game-related tasks, with higher activation in brain regions such as the DLPFC, precuneus, parahippocampal gyrus, and cingulate gyrus (Weinstein and Lejoyeux, 2015). This suggests that different types of addictions, whether short videos, heroin, or online games, share similar attention preferences, with individuals being more sensitive to cues related to their respective addictions (Zhou et al., 2012; Ko, 2014; Lee et al., 2018).

##### 5.1.2.3 Individuals with SVA exhibited higher sensitivity towards negative gains in the OFC

Individuals with SVA demonstrate increased sensitivity to negative outcomes, as shown by the heightened activation of the OFC during loss. In reward-based decision-making, the medial OFC is active, whereas the lateral OFC is implicated in punishment-based decision-making (Zhang M. et al., 2021). Specifically, individuals addicted to short videos exhibited elevated OFC activity when faced with losses, indicating a significant discrepancy between their anticipated rewards from short video content and the actual losses incurred. This suggests that prolonged addiction to short videos may result in amplified reward responses and heightened sensitivity to negative emotions, particularly in situations involving punishment or loss. In contrast, non-addicts may not display the same level of OFC activation in response to short video stimuli as they lack the same level of reward-related expectations. The decision-making process is influenced by risk sensitivity, which dictates whether individuals display risk-seeking or risk-averse behavior, and loss aversion, which refers to the tendency to prioritize potential losses over equivalent potential gains. Individuals often exhibit greater sensitivity to losses than equivalent gains (Asutay and Västfjäll, 2022). Individuals with SVA may exhibit heightened activity in the OFC when making risky decisions because they are more responsive to rewards associated with short video content. By contrast, non-addicts showed lower OFC activation in similar situations, indicating a weaker reward response to short video content. Research has shown that individuals with substance addiction, such as video game and smartphone addiction, exhibit heightened activation in the OFC when faced with scenarios involving loss outcomes (Liu et al., 2023). These findings suggest that short video addiction shares similarities with other forms of psychological and substance addictions, as people with addiction seek greater rewards and are more sensitive to losses, whereas healthy controls do not show significant differences in their brain responses to positive and negative outcomes.

##### 5.1.2.4 Individuals with SVA demonstrate a tendency towards engaging in risky behaviors in short video scenarios

Individuals with SVA exhibit a propensity for risky behaviors. When experiencing losses in short video scenarios, increased activation levels in brain regions such as the FPA and DLPFC were positively correlated with the number of exploding balloons in the task. This suggests that heightened activation in these brain regions may indicate heightened sensitivity to short video-related cues and a tendency towards risky behavior in response to risky stimuli. This positive correlation may indicate a compensatory neurological mechanism in individuals with SVA, in which the brain allocates more resources to processing risk cues in the face of loss, leading to increased risky behavior.

### 5.2 Conclusion

In summary, this study highlights the decision-making behaviors and brain responses of individuals addicted to short videos. It identified heightened sensitivity to short video cues, with significant activation in the OFC and FPA, indicating increased reward values and cognitive processing demands for short video cues. Overactivity toward negative outcomes, as shown by OFC activation in loss scenarios, suggests a heightened sensitivity to negative feedback among individuals with SVA, potentially reinforcing their irrational decision-making. Cognitive control limitations, indicated by FPA and DLPFC activation, suggest that individuals with SVA require additional cognitive effort to navigate complex situations, particularly impulse control, during short video-related tasks. The propensity for risk-taking behavior, reflected in the brain region activation correlating with risky behavior, underscores the high-risk inclination of individuals with SVA, possibly arising from an imbalance between rewards and punishment triggered by short video cues. These findings reveal significant neurofunctional differences in reward processing, loss of sensitivity, and impulse control among individuals addicted to short videos.

This study used fNIRS to examine the neural mechanisms underlying risk decision-making and reward processing in individuals with short video addiction. These findings highlight the OFC, FPA, and DLPFC as critical regions involved in short video addiction, elucidating specific neural pathways. These results lay the foundation for interdisciplinary research on behavioral addiction, precise diagnostics, personalized interventions, and the formulation of public health policies. This study underscores the significant impact of short video stimuli on addiction, suggesting that content delivery strategies on short video platforms (e.g., frequent short-term stimuli) may contribute to addictive behaviors. The implications of this study can inform the development of healthier platform algorithms and management strategies, suggesting that policymakers implement reasonable usage time limits or push notification frequencies to mitigate the adverse effects of excessive use on decision-making processes. Short video addiction is a pressing issue with broad implications for mental health and social functioning. The findings of this study offer a scientific basis for designing neuroscience-informed educational interventions and psychological treatments to address the negative consequences of short video addiction in both individuals and society.

## 6 Limitations and future research

Although this study provides valuable insights, it is important to acknowledge its limitations. The research focused on the correlation between brain activation patterns in individuals addicted to short videos and risky behaviors but did not establish causality. Further investigation is needed to determine whether the hyperactivation of certain brain regions is a cause or consequence of addiction. In addition, the short video cues used in this study may not fully represent the complexity of short video platforms, including their content diversity and visual effects. Potential differences in brain responses to various types of short videos have not been thoroughly explored.

Secondly, our study focused on the functions of the FPA, OFC, and DLPFC in the frontal lobe using fNIRS technology. However, owing to limitations in fNIRS, we were unable to investigate deeper brain regions involved in decision-making, such as the ACC. Future research could combine fNIRS with imaging techniques, such as fMRI, EEG, or MEG, to overcome temporal and spatial resolution constraints, leading to a more comprehensive understanding of the neural mechanisms underlying short video addiction.

The issue of short video overuse and addiction is becoming increasingly severe, affecting a broad range of populations, including not only adolescents but also children and older adults. Although this study confirmed that short video addiction has a specific impact on risky decision-making among college students, with addicted individuals being more prone to making high-risk decisions, it remains unclear whether individuals in other age groups exhibit similar characteristics. In future research, we aim to diversify the participants and broaden the investigation to understand the neural mechanisms of short video addiction across all age groups. This endeavor seeks to establish a biological recommendation for governments and educational institutions to effectively promote the rational use of short videos.

Overall, our study explored decision-making patterns and brain activation levels in individuals with short video addiction, highlighting the associations among short video addictive behaviors, risk propensity, and background cues. These findings contribute to our understanding of the neural mechanisms underlying addiction and offer potential pathways for intervention.

## Data Availability

The original contributions presented in the study are included in the article/supplementary material, further inquiries can be directed to the corresponding author.
